# Trends in socio-demographic characteristics and substance use among high school learners in a selected district in Limpopo Province, South Africa

**DOI:** 10.1186/s12889-024-18927-7

**Published:** 2024-05-27

**Authors:** Linda Shuro, Firdouza Waggie

**Affiliations:** 1 School of Public Health, University of the Western Cape, Robert Sobukwe Rd, Bellville, Cape Town, 7535 South Africa; 2 Faculty of Community & Health Sciences, University of the Western Cape, 14 Blanckenberg Road Bellville, Cape Town, 7535 South Africa

**Keywords:** Substance use, High School Learners, Adolescents, School health, Limpopo, South Africa, Cross-sectional survey, Socio-ecological model

## Abstract

**Background:**

Substance use is an escalating public health problem in South Africa resulting in risky behaviours and poor educational attainment among adolescents. There is a huge battle to overcome substance use among learners as more drugs become easily available with the mean age of drug experimentation reported to be at 12 years of age. It is important to continuously understand the trends in substance use in order to assess if there are positive changes and provide evidence for the development of context-specific effective interventions. This paper outlines the prevalence of substance use among selected high schools in a district in Limpopo province.

**Methods:**

To determine the prevalence of substance use among selected high school learners in a district in Limpopo Province, a cross-sectional school survey of 768 learners was conducted. Data was analysed using SPSS v 26. Descriptive analysis was used to describe the independent and dependent variables and Chi-Square test was used to investigate associations between demographic characteristics and substance use among high school learners.

**Results:**

The most abused substances by learners were alcohol (49%), cigarettes (20.8%) and marijuana (dagga/cannabis) (16.8%). In a lifetime, there was a significant difference (*P* < 0.05) in cigarette smoking with gender, school, and grade; with more use in males (14.2%) than females (7.6%); in urban schools (14.6) than peri-urban (6.7%) and more in Grade 12 (6.4%). There was a significant difference (*P* < 0.05) in alcohol use with more use in Grade 10 (12.6%) and varied use among male and female learners but cumulatively more alcohol use in females (27.7%). Drug use varied, with an overall high drug use in urban schools (20.7%).

**Conclusions:**

Substance use is rife among high school learners in the district and health promotion initiatives need to be tailored within the context of socio-demographic characteristics of learners including the multiple levels of influence such as peer pressure, poverty, unemployment and child headed families. Additional research is required to investigate the factors leading to a notable gradual increase in use among female learners and into the environmental and family settings of learners in influencing substance use.

**Supplementary Information:**

The online version contains supplementary material available at 10.1186/s12889-024-18927-7.

## Background

Substance use and abuse is a major public health concern among female and male adolescents with prevalence varying in different contexts. Drug consumption in South Africa is twice the global average [1]. South Africa is ranked in the top 10 narcotics and alcohol abusers in the world. For every 100 people, 15 have a drug problem and for every 100 Rands in circulation, 25 Rands is linked to substance use [2]. Many schools in South Africa continue to battle with the problem of substance use among learners. The most experimented substances by adolescents in South Africa is tobacco and alcohol [3]. A majority of these adolescents are found in schools. The use of substances at an early age, especially among learners results in negative health and social outcomes such as school dropouts, and early onset of sexual behavior which may lead to teenage pregnancy and sexually transmitted infections [4, 5]. A review of studies on impact of substance use on school performance and public health indicate that use of substances is significantly associated with negative school outcomes such as truancy, low motivation to learn, regular sickness, increasing school abstenteeism, decreasing marks and high chances of skipping school [6–8].

The mean age of drug experimentation in South Africa is 12 years and this is rapidly decreasing [9]. Globally 1 in 4 learners (13–15 years old) had their first smoke before the age of 10 and the percentage of use is greater than 10% for any tobacco product by 13-15-year-old learners [10]. The increased availability and variety of drugs available to South African teenagers is a cause for alarm, for example, marijuana (known as dagga/cannabis), cocaine, glue, methamphetamine known as TIK and whoonga known as “nyaope”-a street name for a mixture of mainly dagga and low-grade heroin [11]. South Africa is also experiencing an up rise in drugs and gangsterism labelled the “twin evils of our time”, especially found among youth in previously marginalized communities. The problem is viewed as an indication of the many socio-economic challenges faced by working class communities [12].

Global initiatives such as the WHO Global School Health initiative promote health promoting schools (HPS) to improve the health of the school community. An HPS is a “school constantly strengthening its capacity as a healthy setting for living, learning and working” [13]. The HPS approach was implemented in South Africa in 1994 in efforts to redress inequalities created during apartheid in the education and health sector and also the policy context was favourable for its acceptance [14]. There are many public health initiatives to address health issues among adolescents in South Africa which are integrated as part of the current health reforms such as re-engineering primary health care, National Drug Master Plan 2013–2017 [15] and the revised Integrated School Health Policy (ISHP). With a focus on school health, the ISHP was launched in 2012, as a collaboration between the Department of Health (DoH) and the Department of Basic Education (DBE) [16] as a framework for the new school health programme (grade 0 to 12 learners), implemented at sub-district level. It is therefore invested at the primary level and aligned to several government commitments such as the United Nations Convention on the Rights of the Child and Bill of Rights of the South African Constitution [17]. The above initiatives require a more integrated approach for effective change and to address the social determinants of substance use among learners [18].

Schools in Limpopo face multiple social challenges that affect effective teaching and learning such as crime and violence, sexual assault/abuse, substance use and bullying [19]. There was a recent outcry for action by learners to the Education Member of Executive Council (MEC) to address these social challenges [20]. A review of prevalence studies [21–25] in Limpopo high schools shows that male learners abuse drugs more than female learners. The review also showed past month low prevalence rates in rural high schools but with progression of studies and lifetime use, a gradual increase in prevalence rates in schools was noted. Some of the contributing factors to substance use noted include more access to finances by the males, the presence of liquor stores near the learners’ homes; certain demographic characteristics such as being male, urban versus rural learners; substance use among parents and friends. The major determinants of alcohol use found in students include, “gender, age, ever having smoked a cigarette, ever damaged property, walking home alone at night, easy availability of alcohol, thinking alcohol use was wrong, attending religious services and number of friends who used alcohol” [21]. A similar study identified the following five community level factors linked to use of home prepared alcohol by learners: i) subjective adult norms around substance use in the community, ii) negative opinions about one’s neighbourhood, iii) perceived levels of adult antisocial behavior in the community, iv) community affirmations of adolescents, and v) perceived levels of crime and violence in the community (derelict neighbourhood)” [26]. In one district in Limpopo, learners identified alcohol, tobacco, marijuana, petrol, glue and jeyes fluid mixed with spirit as the commonly used substances and other learners experimented on heroin and cannabis as they had friends with access to the drugs in town [27] which is consistent with other studies [28, 29]. Youth in Limpopo are engaged in different substances (tobacco, alcohol, hard core drugs) with cannabis, inhalants, bottled wine, home, and commercially brewed beer as commonly abused substances [30]. This highlights the need for more monitoring studies to review the escalating situation of substance use among learners to create a wider data baseline for evidence-based initiatives. One of the research sessions at the 47th annual meeting of the Society for Epidemiologic Research on to tobacco and smoking showed that continued publication of health effects leads to reduction in smoking [31].

This study adds on to recent prevalence studies on substance use in high schools and adolescents in Limpopo province. Additionally, it contributes to baseline information which assists in the development of evidence-based initiatives. In line with the aims of international surveys [32] from which this study adopts, the findings support reporting of comparable data on drug use trends in Limpopo as well as having data from 1 of the 6 districts helps comparison within the province and supports targeted intervention and not a one size fits all approach. The main researcher was involved in anti-substance use clubs in some schools in Limpopo as part of health promotion and in light of many existing policies, it is worrying to note a gradual increase in prevalence rates of substance use amongst learners noting the gap between what is on paper and actual implementation (Lenkokile, 2016; Madikane, 2018; Mokwena et al., 2020). This study was an important process in Limpopo focused at providing current evidence towards developing effective context specific anti-substance use initiatives in high schools. The aim of this study was therefore to determine the prevalence of substance use among selected high school learners in schools in one district in Limpopo Province.

## Methods

### Study design

A cross-sectional survey (quantitative) was conducted among 768 high school learners from four high schools in the district.

### Study population and sampling

The study population was all high school learners (N-13 244) enrolled in the period 2019–2020, Grade 8 to 12 [33]. Fifteen high schools within the Polokwane circuit were stratified into two strata according to socioeconomic and geographical divide: Urban and Peri-Urban. Simple random sampling was used to select two schools from each stratum. Once the four schools were identified, simple random sampling was used to select one class each from Grade 8 to 12 for participation in the cross-sectional survey. Consideration was taken to include the whole class so that learners are treated equally and excluding some students could affect anonymity perceptions and lead to disturbances [32]. However, due to the pressure of the announcement of the lockdown due to Covid 19 in March 2020 and schools closing, random sampling of classes was a bit limited to available classes in each grade.

### Research sites

The study took place in four public high schools in the Polokwane circuit, Capricorn district, Polokwane local Municipality. The two peri urban schools selected are in the Seshego cluster on the north-west outskirts of the Polokwane city which is divided into 8 residential zones. Seshego is diverse with both extremes (poverty and wealth) located about 5kms from the CBD and most people must commute to the city for work. The urban schools are found more adjacent to the Polokwane city in Nirvana and Flora Park (formerly “coloured” and white” suburbs) but now quite diverse [34]. Polokwane is found in the Limpopo Province, South Africa. Limpopo is a rural province with 5 district municipalities: Capricorn, Sekhukhune, Waterberg, Vhembe, and Mopani. Within the Capricorn district are four local municipalities: Polokwane, Blouberg, Molemole and Lepelle-Nkumpi [35].

### Data Collection

The school management and the heads of department for Life Orientation were instrumental to grant permission to conduct the survey and to randomly select one class per each grade to participate in the schools. The questionnaire was distributed to the learners to fill in and the researcher was present to explain the purpose of the research and address any clarifications. The survey took place in March 2020, a few weeks, and days before the national lockdown due to COVID 19. The self-administered questionnaire used in this study, is a modified instrument adapted from the UNODC Global Assessment Programme on Drug Abuse (GAP) Toolkit questionnaire on Conducting School Surveys on Drug Abuse. This tool is deemed valid and reliable as it was used and adapted in previous studies [36–38]. The original questionnaire was developed to build local level capacity among member states to collect data that can guide reduction activities in schools and therefore better fits the purpose for this study. The questionnaire was adapted to the local context using SA based terms and removing terms not relevant to the local context. A pilot study was conducted in a different circuit and adjustments made to the questionnaire and the process of data collection.

### Ethical considerations

Permission to conduct the study was granted by the Limpopo Department of Education (Ref: 2/2/2) and the University of Western Cape (HS19/9/12). Information sheets, consent, and assent forms to participate in the study and seek permission from a guardian or parent were given to the learners prior to the data collection date. The researcher went with the invitations, information sheets and consent forms to the education circuit and these were sent to each school via the circuit office. The researcher also went with copies of the information sheets for the parents and learners to each school before the data collection. Therefore, the learners were informed of the study by providing them with an information sheet and explaining the purpose of the research and what is expected of them. An information sheet was also provided for the parents or guardians. Parents received information sheets and parental consent sought for learners to participate in the school survey. The signed forms from the parents/guardians were collected before administering the questionnaire to learners. Before administering the questionnaire, an explanation was provided again and learners above 18 received the consent forms and assent forms for learners under 18 to agree to participate in the study once they fully understood the purpose of the research.

### Data analysis

Microsoft Excel was used to capture the data and exported to IBM SPSS v 26 for analysis to obtain baseline information about substance use in high schools (Briggs, 2016). Descriptive analysis was used to describe the independent and dependent variables using percentages, means, and standard deviation and inferential analysis such as correlation between sociodemographic characteristics and substance use, was used as well [39]. Percentages of gender, age, school, grades, level of parent’s education and person living with the learner were described. The frequency of substance use (alcohol, cigarette smoking and drugs) was presented to show lifetime, during the last 12 months and past 30 days (previous month) substance use. The Chi-Square test was used to investigate associations between demographic characteristics and substance use (cigarette smoking, alcohol use and drug use). The different p-values of less than 0.05 at a 5% significance level obtained, suggested that either grade, school, and gender have an influence and the differences on substance use depending on the substance. Percentages on awareness of substances, disapproval of substance use, friends who used substances, access to substances, perceived risk and associated behaviours under influence of substance use.

## Results

### Demographic characteristics

Seven hundred and sixty-eight (*N* = 768) learners from four high schools participated in the survey. 54.2% (*n* = 416) were female and 45.8% (*n* = 352) male learners, with a mean average age of 16 years. There were 286 participants from two schools in the peri-urban (school 2 and 3) and 482 participants from the urban environment (school 1 and 4). The percentage of grades was distributed proportionally with a slightly increased percentage among the Grade 10s. The break-down of the participants is represented in Table 1. The percentage of participation in each grade in each school varied due to class size variation. For purpose of this article, only geographical location (urban and peri-urban), gender, age and school are reported in relation to substance use.

**Table 1:** Biographical information of the Learners (*N* = 768)

		*n*	%
School	School 1 (urban)	235	30.6
	School 2 (peri-urban)	108	14.1
	School 3 (peri-urban)	178	23.2
	School 4 (urban)	247	32.2
Grade*	Grade 8	153	19.9
	Grade 9	150	19.5
	Grade 10	163	21.2
	Grade 11	155	20.2
	Grade 12	147	19.1
Gender	Male	352	45.8
	Female	416	54.2
Age	12	1	0.1
	13	27	3.5
	14	129	16.8
	15	148	19.3
	16	135	17.6
	17	122	15.9
	18	89	11.6
	19	62	8.1
	20	26	3.4
	21	13	1.7
	22	1	0.1
	23	1	0.1
	Not indicated	14	1.8

### Perceived availability and awareness of substances

In the four participating schools, learners responded to availability of several substances with cigarettes indicated as the most easily accessible substance 46.5% (*n* = 357) and mandrax the least accessible (see Table 2). The results indicate a wide variety of substances available to learners.

**Table 2:** Perceived availability of substances

	Impossible	Very difficult	Fairly difficult	Fairly easy	Very easy	Don’t know	Not indicated	Total
Cigarettes	18.8	15.8	7.9	23.3	23.2	6.5	4.6	100%
A small bottle of spirits (ca. 35 cl)	27	21.5	10.2	14.1	12.4	11.2	3.8	100%
Marijuana/ Dagga (cannabis, pot, grass)	28.1	25.3	9	12	10.8	9.9	4.9	100%
Nyaope/Whoonga	31.9	27.2	8.7	9.5	5.3	12.8	4.6	100%
Amphetamines (pills, bennies, speed)	36.1	25.1	10.7	4.6	3.8	15.2	4.6	100%
Tranquillisers or sedatives	36.7	24.6	10.3	4.4	3.4	15.2	5.3	100%
Cocaine/Crack	38.5	27	8.7	3.9	3.3	13.7	4.9	100%
Mandrax	38.9	27.6	8.7	2.9	2	15.3	4.6	100%
Ecstasy	38.3	26.8	9.2	2.7	2.3	15.5	4.9	100%
Heroin (smack, horse)	38.4	26.6	7.8	4.2	2.7	15.6	4.7	100%
Solvents or inhalants (glue etc.)	34.6	22.8	7.6	6.6	8.7	14.6	5	100%

When learners were asked if they ever heard of the drugs listed on the questionnaire, learners had mostly heard of marijuana 72.1% (*n* = 554), nyaope 69.8% (*n* = 536), and least heard of ecstasy 22.3% (*n* = 171) and other drugs 22.5% (*n* = 173). Learners went on to mention the other drugs which included petrol, vape, tretamines and names that seemed mostly to be street names such as weed, Bluetooth, globe, flakka, hubbly, hashishka, cat, lollipop, ice pace and soil pill.

### Trends in cigarette smoking and alcohol use

The overall lifetime prevalence of cigarette smoking among the learners was 20.8% (split according to the number of occasions (from 1 to 2 times to 40+) as seen in Table 3). 11.4% of the learners responded to have smoked during the last 12 months. In the last 30 days there was an overall prevalence of 7.1% further broken down by number of occasions. There was a high lifetime overall utilisation of alcohol with 49% of the learners having drunk alcohol (split according to the number of occasions (from 1 to 2 times to 40+) in Table 3. 37.1% indicated alcohol consumption during the last 12 months with 13.7% who did not indicate. In the last 30 days the overall prevalence was at 20.9% with 16.3% who did not indicate. 9.6% of the learners indicated that they had five or more drinks in a row at least once and 4.4%, 10 or more times in the last 30 days.

**Table 3:** Trend in use- Number of occasions of alcohol use and cigarette smoking:

Cigarette Smoking (%)			
Number of Occasions	Lifetime	Last 12 months	Last 30 days
Never 1–2 3–5 6–9 10–19 20–29 40+ Not Indicated	74.9 8.7 2.6 2 2.3 1 4.2 (20.8) 4.3	73.5 4 2 1.3 0.8 0.4 2.9 (11.4) 15.1	76.5 2.3 1.6 0.4 0.4 0.4 2 (7.1) 16.4
**Alcohol Use (%)**			
**Number of occasions**	**Lifetime**	**Last 12 months**	**Last 30 days**
Never 1–2 3–5 6–9 10–19 20–39 40+ Not Indicated	45.2 18.2 9.8 5.3 3.5 3.5 8.7 (49) 5.7	49.3 14.1 7.6 4.7 4 3.3 3.4 (37.1) 13.7	62.9 9.1 3.5 2.9 2 0.9 2.5 (20.9) 16.3

An attempt was made to establish whether the learners’ socio-demographic characteristics were associated with cigarette smoking and alcohol use in a lifetime. The results as shown in Table 4 shows Chi-square test with a P- value of 0.000 at a 5% significance level suggesting that grade, school and gender have an influence on lifetime cigarette smoking. Schools in the urban area had an overall higher prevalence of cigarette smoking (14.6%) than schools in the Peri-Urban (7%) as cumulative effect. The results also show a significant difference in use for cigarette smoking by gender with more males (14.2%) than females (7.6%). The results in Table 4 also shows Chi-square test with a P- value of 0.001 for grade and 0.000 for gender suggest an association between lifetime alcohol use and these two socio-demographic characteristics with most alcohol use found to be in grade 10 (12.8%) and least in grade 8 (8%) as a cumulative effect. The differences in use among male and female learners varied with the number of occasions with overall high use in females (27.7%). There was no significant difference in alcohol use with schools.

**Table 4:** Association between sociodemographic characteristics of learners and lifetime cigarette smoking and alcohol use

Variables	Attribute	Cigarette Smoking		Alcohol Use	
		Overall use %	P-Value	Overall use %	P-Value
School	Urban	14.6	*P* = 0.000 Χ^2^ = 55.027 df = 18	31.1	*P* = 0.203 Χ^2^ = 22.681 df = 18
	Peri-Urban	7		20.9	
Grade	Grade 8	3.7	*P* = 0.000 Χ^2^ = 59.223 df = 24	8	*P* = 0.001 Χ^2^ = 52.845 df = 24
	Grade 9	2.2		9	
	Grade 10	5.3		12.8	
	Grade 11	4.1		11.4	
	Grade 12	6.4		10.8	
Gender	Male	14.2	*P* = 0.000 Χ^2^ = 43.640 df = 6	24.5	*P* = 0.000 Χ^2^ = 24.887 df = 6
	Female	7.6		27.7	

Results showed that the age at first use of alcohol (beer, wine) and cigarette smoking was quite low at 13 years or less with a significant percentage even below 15 years of age. An age of 13 years or younger was taken as an indicator of early onset. At the age of 13 years or younger: 18.5% (*n* = 142) of the learners had drunk beer, 18.8% (*n* = 144) drank wine and 11.1% (*n* = 85) had smoked cigarettes. There was a percentage decline in use with increasing age.

### Trends in drug use

#### Learners who tried drugs

When asked if they had ever tried the listed drugs on the questionnaire, 19.3% male learners and 13.9% of the female learners said “Yes” to marijuana. The results for the other drugs had lower percentages which varied but indicate more males had tried out substances than females.

#### Lifetime use of drugs, during 12 months and last 30 days

**Fig. 1:**
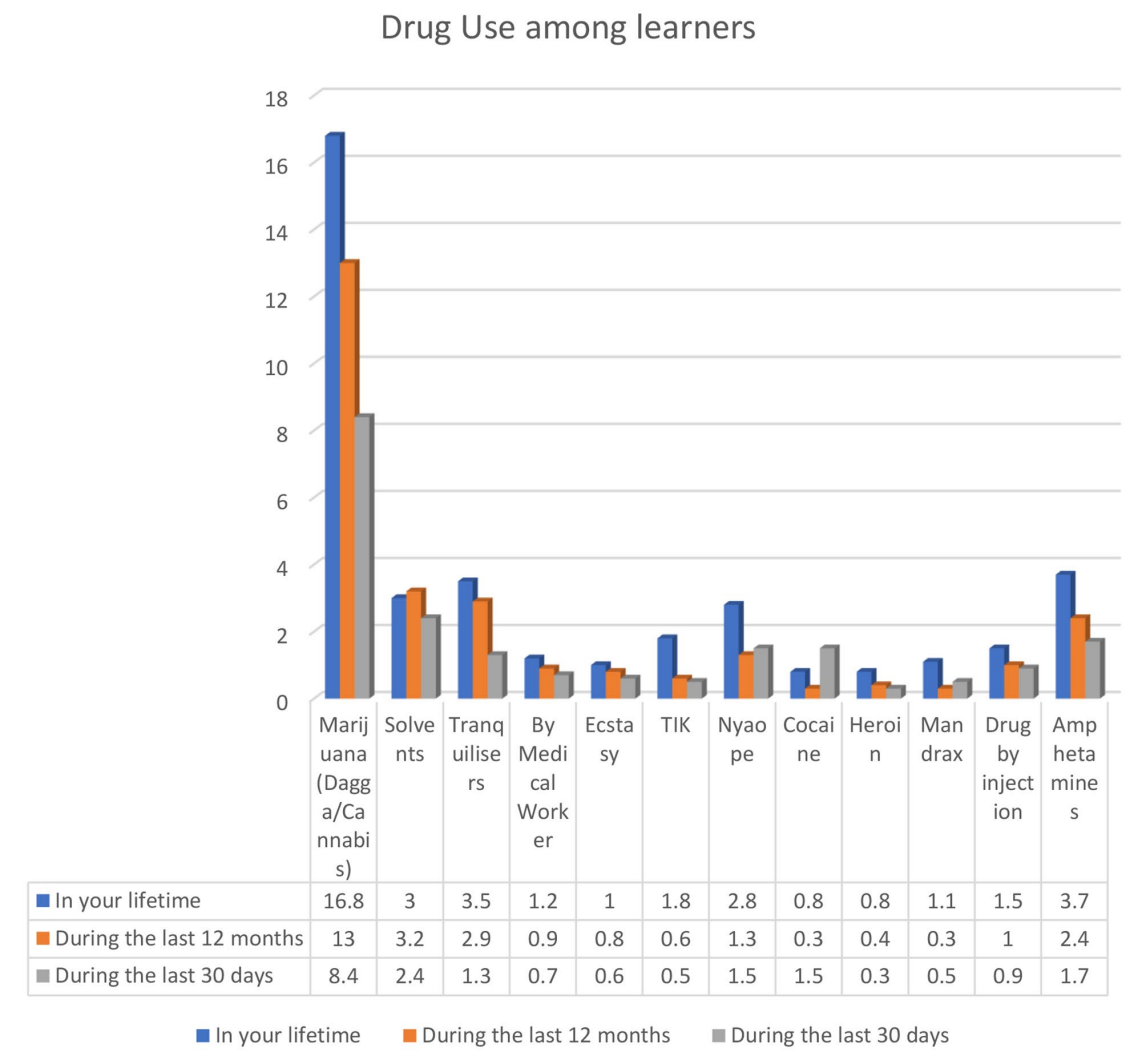
Drug use- Lifetime, During last 12 months and last 30 days

Lifetime use of drugs varied with the type of drugs (see Fig. 1). A percentage overall of 16.8% of the learners had used marijuana in a lifetime, 13% in the last 12 months, and 8.4% in the last 30 days (see Fig. 1). Results also showed that the most common drug first tried was marijuana (dagga) (12.1%).

The Chi-square test was applied to investigate the association between socio-demographic characteristics and lifetime use of drugs. There was a significant association between school and the use of drugs prescribed by medical workers (*P* = 0.03), ecstasy (0.01), nyaope/whoonga (0.025) and mandrax (0.03) with higher use in urban (20.7%) compared to peri-urban (16.9%) schools. There was an association between grade and the use of marijuana (dagga) (0.000) with overall high use from Grade 10 to 12 and gender on the use of marijuana (0.000) and nyaope (0.018) with more males (25.1%) than females (14.1%).

### Risk of substance use

46% (*n* = 352) of learners perceived great risk with smoking one or more packs of cigarettes per day, 40% (*n* = 307) perceived great risk by having four or five drinks in a row nearly every day, 24% (*n* = 183) of the learners perceived smoking cigarettes occasionally as a great risk but closely 22% (*n* = 169) perceived no risk with smoking occasionally, 19% (*n* = 147) no risk with having one or two drinks nearly every day and 16% (*n* = 125) no risk with trying marijuana.

## Discussion

This study investigated the prevalence of substance use among high school learners and highlighted how certain socio-demographic characteristics such as gender, grade, and school influence substance use patterns.

Notably, our findings reveal gender disparities in substance use. Whilst alcohol use varied with the number of occasions among female and male learners, there was an overall high alcohol use in females (27.7%) compared to their male counterparts (24.5%). It is essential to recognize that our study, primarily focused on prevalence, and did not delve into the determinants of substance use specifically within gender groups. Nonetheless, contextual factors such as poverty and gender-based violence prevalent in South African communities may contribute to the elevated substance use rates observed among female learners. Additionally, delays in accessing social assistance could potentially exacerbate risky behaviors among this demographic. Additional research is needed into factors influencing a gradual increase in uptake among female learners which was beyond the scope of this study.

The study also shows further gender disparity with male learners demonstrating higher levels of experimentation and use of drugs, particularly marijuana and nyaope (whoonga). This trend aligns with existing research, which consistently indicates a higher prevalence of substance abuse among male learners. A review of past and present prevalence studies conducted in Limpopo high schools corroborates this observation, with multiple studies consistently reporting higher rates of drug abuse among male learners [21–26]. Assumptions from observation can be made that this could be linked to societal settings and friends in which boys “hang out” with more than girls and may have ease of access to drugs. Males tend to be found more on the corners of streets, shops and stay out late. The phenomenon of having more male users than females is found in many prevalence studies [20, 29, 48–50] suggesting the need for male-oriented initiatives. To show the magnitude of the problem *“Harmful use of alcohol is accountable for 7.1% and 2.2% of the global burden of disease for males and females respectively”* [51]. However, several studies are beginning to show that there is little difference in use between genders [55]. This suggests the need for gender-specific initiatives to ensure effective programs among adolescents.

The results of the present study indicate that substance use is rife in both peri-urban and urban environments among high school learners in the district. In a lifetime, cigarette smoking (20.8%), alcohol (49%), and marijuana (16.8%) were identified as the commonly used substances among the learners, mirroring trends observed in past studies conducted in Limpopo and Sub-Saharan Africa. A baseline study among youth conducted in Limpopo in 2013 showed percentage use of inhalants at 39%, marijuana (49%) and alcohol (54,8%) as the most used substances [30]. Similarly, a systematic review of 27 studies in Sub-Saharan Africa among 143 201 adolescents shows that alcohol (32.8%), tobacco products (23.5%), khat (22%) and cannabis (15.9%) were the most commonly used substances [53].

Despite these similarities, the present study indicates a decrease in the percentage use of these substances compared to past studies. This discrepancy could potentially be attributed to the present study’s focus on selected high schools within a district, as opposed to previous studies that may have had broader sampling across the entire province or region. Nonetheless, overall this data is comparable to a certain extent to results of national surveys that have been conducted using a similar instrument which show trends in use in a lifetime, annually and in the past month and correlations across demographic characteristics and substance use. Examples include the annual drug national survey of 2020 (Monitoring the Future) in the United States [38] and an older survey in Kenya on patterns of drug use in public secondary schools [36]. Therefore, the findings add to a wider data baseline for evidence-based initiatives and more specific to Limpopo towards evidence-based context-specific anti-substance use initiatives in high schools.

A worrying phenomenon observed in this study and other previous studies is the decreasing age of onset of substance use at the age of 13 years or less. Despite the legal restrictions in South Africa setting the minimum age purchasing alcohol and cigarettes at 18, adolescents are gaining access to these substances, as indicated by the study findings. The findings are consistent with the study conducted in Limpopo in 2013, which shows the age of first use of cannabis/marijuana as early as 10 years or less [29]. There have been policy discussions to amend the age to 21 but this may seem not to be effective considering that alcohol is easy to access by a 13-year-old or younger suggesting a “*thriving illegal market”* [40]. A similar finding by the Southern Africa Alcohol Policy Alliance (SAAPA) showed that 12% of those under 13 years were said to have drunk alcohol in the past month and 25% of young people binge drinking [40]. The rise in underage use may also be linked to the ongoing alcohol advertising which is prominent in the neighbourhoods and entices adolescents. With mixed views on the introduction of the new Limpopo tobacco bill, it’s crucial to monitor any changes in adolescent access to substances. Calls for the bill to enforce strict measures on the sale of tobacco products to minors highlight the urgency of addressing this issue [52].

According to the WHO Global status report on alcohol and health [41], *Alcohol is the leading risk factor for premature mortality and disability among those aged 15 to 49 years, accounting for 10% of all deaths in this age group.* This highlights a huge public health challenge and the need for preventive strategies that target lower grades before the onset of substance use. Whilst learners are aware of the different types of substances there is still a significant number of learners who do not perceive the risk of smoking and drinking occasionally and trying marijuana. To improve the perceived risk associated with substance use among learners, as part of integration in education, consistent awareness programmes in all the subjects of the curriculum could assist in improving the level of perceived risk and not limited to the Life Orientation subject in the school curriculum only as recommended that health education should be established in all school topics [42]. . This approach aligns with recommendations from the U.S. Department of Health & Human Services [56], which emphasize the significance of prevention programs at different life stages and involving the community.

The increased lifetime utilisation of alcohol, cigarettes, and marijuana is linked to the ease of access of these substances as first experimental substances, providing a gateway for the introduction of other substances [30]. In line with the ecological model on determinants of health, some of the multiple factors for increased use and availability of marijuana among the learners could be that South Africa is ranked among the countries in the region where cannabis cultivation and production occur to a large extent and marijuana use is legalised for adults to cultivate and smoke in their homes. A contributing factor to use by learners is substance use by parents or adults they live with. In 2017, 3.8% of the global population aged 15 to 64 years used cannabis at least once and cannabis use increased significantly between 2010 and 2017 in Africa [46]. Cannabis (marijuana/dagga) is highly used globally with approximately 3.8% between 15 and 64 years, about 188 million people having used it once or more times in 2017 (UNODC, 2019). Cannabis is ranked among the most used substances among adolescents attributed to its ease of access and a low and drop in the percentage of the perceived risk of using it, as evidenced in Western countries (UNODC, 2018; UNODC, 2014; UNODC, 2021).

In terms of geographical determinants of substance use and abuse, there were higher percentages of cigarette smoking and drug use in the urban schools compared to peri-urban schools. There is a diverse group of learners attending schools in urban areas coming from different areas including from the peri-urban and rural areas. The majority of the learners commute to attend schools and are exposed to access and use of substances when traveling which contributes as a factor to the ongoing public health concern of substance use among adolescents. This phenomenon of learners commuting long distances is also highlighted by a study in New York where older students, girls, and higher attaining students are likely to commute to distant schools despite schools close to them [54]. As a result, the road to school exposes learners to many risky situations including access to substances. A collaboration between the Department of Education and the Department of Transport should exist to ensure safe transport systems for learners whilst, at the same time, more work needs to go into improving local schools working together with the local municipality.

There is a need for policy coherence in all sectors to address substance use especially in the health, education, justice, transport, trade and social development sectors. Policies in the trade sectors which ensure strict adherence to the sale of substances, harsher sentences in the justice sector for the illegal market, improvement in quality of local schools and improved learner transport, if adequately implemented can curb the access and exposure of substances among minors. Key policies like the National Drug Master Plan, which aims for a drug-free society through collaboration with other national departments such as health, justice, and education, need strengthening and universal implementation, including in all schools [47]. While numerous health-oriented public policies exist, there’s a noticeable gap in their implementation, highlighting the necessity for increased resources allocated toward their effective execution [43].

The revised ISHP has a component on health education on prevention of substance use. If implemented in all schools, it has a potential to reach learners and raise awareness at an early age and across all phases (foundation, intermediate and senior) on the dangers of use and may contribute more effectively to the reduction of substance use and early onset [44]. The School Safety Programme which derives itself from many of the policies including the South African Constitution, School Health Promotion, and Children’s Justice Act should also be strengthened to assist in the prevention and control of drugs in schools. The programme has a component of building capacity among school stakeholders [45]. Training should be escalated to all including educators to assist with the timely identification and intervention of learners who use drugs. Parents or guardians need to be included in the training and implementation process for prevention to support behavior change among the learners.

### Limitations

There are two major limitations in this study that could be addressed in future research. Firstly, the analysis was restricted to specific socio-demographic variables, overlooking the potential influence of parents’ educational attainment and the composition of the learners’ living environment. Due to constraints in resources and time, this study did not delve into the association between substance use and these factors. However, exploring the impact of parents’ level of education and the presence of individuals residing with the learners could offer a more comprehensive understanding of the underlying determinants of substance use. Future research endeavors should prioritize investigating these associations to enrich the existing knowledge base.

Secondly, the selection of classes was not randomized but based on availability, primarily due to logistical challenges exacerbated by the unforeseen onset of the Covid-19 pandemic and subsequent lockdown measures. Schools were compelled to adapt their operations swiftly, hindering the feasibility of a randomized sampling approach. Despite this limitation, the selected classes adequately represented the target population and facilitated the attainment of the study’s objectives. It is important to note that the deviation from randomization was a pragmatic adjustment necessitated by external circumstances and did not compromise the integrity or validity of the research findings.

Addressing these limitations in future studies will enhance the comprehensiveness of investigations into the complex dynamics of substance use among learners.

## Conclusions

The findings of the study indicate that substance use is rife in high schools and utilization varies with sociodemographic characteristics. A more robust approach should be implemented which supports consistent health education integrated within all subjects of the curriculum from an early age. This should explore interactive means to reach the different social and educational platforms for learners to be informed, perceive the risk of substances from an early age and receive support. Findings also suggest the need for tailored health promotion programmes depending on the demographics of the learners (gender, grade and location of school) while also addressing the social determinants of substance use. One size does not fit all. The findings of this study contribute to the body of updated evidence on the prevalence of substance use in Limpopo Province among high school learners.

### Electronic supplementary material

Below is the link to the electronic supplementary material.

Supplementary Material 1

## Data Availability

The datasets used and/or analysed during the current study are available from the corresponding author on reasonable request.

## Abbreviations

CDA: Central Drug Authority
DBE: Department of Basic Education
DOE: Department of Education
DOH: Department of Health
DSD: Department of Social Development
DCGTA: Department of Corporate Governance and Traditional Affairs
HIV: Human Immuno-deficiency Virus
HOD: Head of Department
HPS: Health Promoting Schools
ISHP: Integrated School Health Policy
NSSF: National School Safety Framework
PHC: Primary Health Care
SAMRC: South African Medical Research Council
SANCA: South African National Council on Alcoholism and Drug Dependence SACENDU: South African Community Epidemiology Network on Drug Use
Stats SA: Statistics South Africa
SGB: School Governing Body
SMT: School Management Team
TIK: Crystal Methamphetamine
UNODC: United Nations Office of Drugs and Crime
UNFPA: United Nations Population Fund
UNICEF: United Nations Children’s Fund
WHO: World Health Organisation
YRBS: Youth Risk Behaviour Survey
