# Supplementary material for: Trends in socio-demographic characteristics and substance use among high school learners in a selected district in Limpopo Province, South Africa
Source: BMC Public Health. 2024 May 27;24:1407. doi: 10.1186/s12889-024-18927-7 (PMC11129414; doi:10.1186/s12889-024-18927-7)
Supplement: Supplementary file 1 — Supplementary Material 1 [file 12889_2024_18927_MOESM1_ESM.docx]

Supplementary Material: Trends in socio-demographic characteristics and substance use among high school learners in a selected district in Limpopo Province, South Africa

Additional tables on perceived risk and disapproval.

*Table 1 Disapproval of substance abuse*

|  | **Don't disapprove** | **Disapprove** | **Strongly disapprove** | **Don't Know** | **Not indicated** | **Total** |
| --- | --- | --- | --- | --- | --- | --- |
| Smoking 10 or more cigarettes a day | 30% | 22% | 33% | 8% | 7% | 100% |
| Having five or more drinks*) in a row each weekend | 30% | 28% | 26% | 12% | 4% | 100% |
| Trying marijuana/dagga (cannabis, pot, grass) once or twice | 27% | 27% | 30% | 11% | 5% | 100% |
| Smoking marijuana/dagga occasionally | 28% | 24% | 33% | 10% | 5% | 100% |
| Smoking marijuana/dagga regularly | 23% | 23% | 40% | 9% | 5% | 100% |
| Trying heroin (smack, horse) once or twice | 19% | 23% | 41% | 11% | 6% | 100% |
| Trying tranquillisers or sedatives (without a doctor or medical worker telling you to do so) once or twice | 21% | 22% | 40% | 12% | 5% | 100% |
| Trying Ecstasy or any amphetamine (pill, speed) once or twice | 20% | 24% | 39% | 12% | 6% | 100% |
| Trying cocaine or crack once or twice | 20% | 23% | 41% | 10% | 5% | 100% |
| Trying ecstasy once or twice | 20% | 23% | 40% | 11% | 5% | 100% |
| Trying solvents or inhalants (glue etc) once or twice | 18% | 23% | 43% | 11% | 5% | 100% |

*Table 2 Perceived risk of substance abuse*

|  | No risk | Slightly risk | Moderate risk | Great risk | Don't know | Not indicated | Total |
| --- | --- | --- | --- | --- | --- | --- | --- |
| Smoke cigarettes occasionally | 22% | 31% | 17% | 24% | 4% | 3% | 100% |
| Smoke one or more packs of cigarettes per day | 10% | 17% | 18% | 46% | 6% | 4% | 100% |
| Have one or two drinks*) nearly every day | 19% | 22% | 22% | 28% | 5% | 5% | 100% |
| Have four or five drinks*) in a row nearly every day | 11% | 16% | 21% | 40% | 6% | 6% | 100% |
| Have five or more drinks*) in a row each weekend | 12% | 15% | 21% | 37% | 7% | 8% | 100% |
| Try marijuana/ dagga (cannabis, pot, grass) once or twice | 16% | 19% | 16% | 34% | 8% | 7% | 100% |
| Smoke marijuana/dagga occasionally | 14% | 15% | 18% | 39% | 8% | 5% | 100% |
| Smoke marijuana/dagga regularly | 10% | 11% | 17% | 47% | 8% | 7% | 100% |
| Try an amphetamine (pill form, speed) once or twice | 9% | 14% | 18% | 42% | 10% | 6% | 100% |
| Take amphetamines regularly | 7% | 9% | 14% | 51% | 11% | 6% | 100% |
| Try cocaine or crack once or twice | 9% | 11% | 20% | 45% | 11% | 4% | 100% |
| Take cocaine or crack regularly | 7% | 8% | 16% | 52% | 11% | 6% | 100% |
| Try ecstasy once or twice | 9% | 9% | 22% | 43% | 12% | 5% | 100% |
| Take ecstasy regularly | 7% | 8% | 15% | 52% | 12% | 6% | 100% |
| Try solvents or inhalants (glue etc) once or twice | 8% | 13% | 20% | 42% | 11% | 5% | 100% |
| Take solvents or inhalants regularly | 8% | 9% | 14% | 53% | 11% | 5% | 100% |
